# Review of nanostructured devices for thermoelectric applications

**DOI:** 10.3762/bjnano.5.141

**Published:** 2014-08-14

**Authors:** Giovanni Pennelli

**Affiliations:** 1 University of Pisa, Dipartimento di Ingegneria dell’Informazione, Via Caruso 16, I-56122 Pisa, Italy

**Keywords:** nanofabrication, nanostructures, silicon nanowires, thermoelectricity

## Abstract

A big research effort is currently dedicated to the development of thermoelectric devices capable of a direct thermal-to-electrical energy conversion, aiming at efficiencies as high as possible. These devices are very attractive for many applications in the fields of energy recovery and green energy harvesting. In this paper, after a quick summary of the fundamental principles of thermoelectricity, the main characteristics of materials needed for high efficiency thermoelectric conversion will be discussed, and a quick review of the most promising materials currently under development will be given. This review paper will put a particular emphasis on nanostructured silicon, which represents a valid compromise between good thermoelectric properties on one side and material availability, sustainability, technological feasibility on the other side. The most important bottom-up and top-down nanofabrication techniques for large area silicon nanowire arrays, to be used for high efficiency thermoelectric devices, will be presented and discussed.

## Introduction

The thermoelectric (TE) effect, known since the 19th century, offers an interesting perspective for the direct conversion of heat in electrical power, and vice versa. Given a thermal gradient, a thermoelectric generator (TEG) is capable of converting heat into electrical power even with small temperature differences. TEGs are simple, compact, robust and very reliable because they contain no moving mechanical parts. For all these reasons, TEGs are attractive for a large variety of applications, in particular in the fields of energy recovery and green energy harvesting. For example, they can be used as alternative to photovoltaic cells [1–2], or together with advanced photovoltaic cells [3–4], for the conversion of solar energy into electrical power.

Several industrial processes need a large amount of heat, which is wasted and dispersed in the environment at the end of the productive cycle because it cannot be converted by conventional thermodynamic systems (as turbines or steam engines). All these industrial processes could conveniently exploit TEGs for recovering most of this otherwise wasted heat. Several studies are dedicated to the development of modules, based on TEGs, for exhaust heat recovery in cars [5–6]. Several car maker companies shows a growing interest in these modules, to be applied for the production of electrical power in hybrid cars and/or for the supply of the on-board car electronics and sensors. One more application field of TEGs is energy scavenging. For example, body heat can be used for powering wearable sensor systems or, eventually, for powering personal electronics as mobile phones. It must be mentioned that TEGs are also used as heat pumps and/or as generators in domestic plants for air conditioning or heating.

However, with the current state-of-the-art technology, the applications of TEGs are limited because their thermal to electrical conversion efficiency is still quite low. This is mainly due to the limitations of the materials currently available for the fabrication of thermoelectric generators. A great research effort is still needed for the development of materials with thermoelectric properties capable of conversion efficiencies greater than 10%. In particular, one of the most limiting factors that reduce the efficiency is the heat diffusion through the TEG for thermal conduction: Most of the heat passes through the generator and it is wasted on the cold side without being converted in useful electrical power. Thus, one of the main target of research efforts in thermoelectricity is to develop materials with a very low thermal conductivity, while still maintaining a high electrical conductivity. In this way, Joule heating, which is an irreversible process, is reduced and, furthermore, high electrical currents can be delivered to the external load.

In this review, first of all the general principles of thermoelectricity will be summarized (section I “Principles of thermoelectricity”). Section II, “Materials for thermoelectricity”, will describe the principles for the optimization of TEG efficiency. Then, the most common thermoelectric materials currently available will be briefly illustrated. Section III will show the advantages of nanostructured materials, with respect to bulk ones, for the purposes of thermoelectric conversion. The development of materials for a large scale application of thermoelectric generation should consider a trade-off between optimal thermoelectric properties on the one hand and material availability, cost, sustainability and technological aspects [7] on the other hand. In this respect, silicon is the second most abundant element on the surface of Earth (after oxygen), it is a very sustainable and biocompatible material. Furthermore, due to its pervasiveness in the electronic market, silicon is one of the best known materials both from the physical and the technological point of views, and it is at the center of a worldwide manufacturing infrastructure. Thermoelectric applications of silicon are currently limited by its high thermal conductivity (148 W/mK). However, several studies have observed a strong reduction of thermal conductivity in rough silicon nanowires [8–10]. For this reason, section IV is dedicated to the review of the main techniques currently investigated for the fabrication of silicon nanostructures that can be integrated in devices for thermoelectric generation.

## Review

### Principles of thermoelectricity

**Figure 1:**
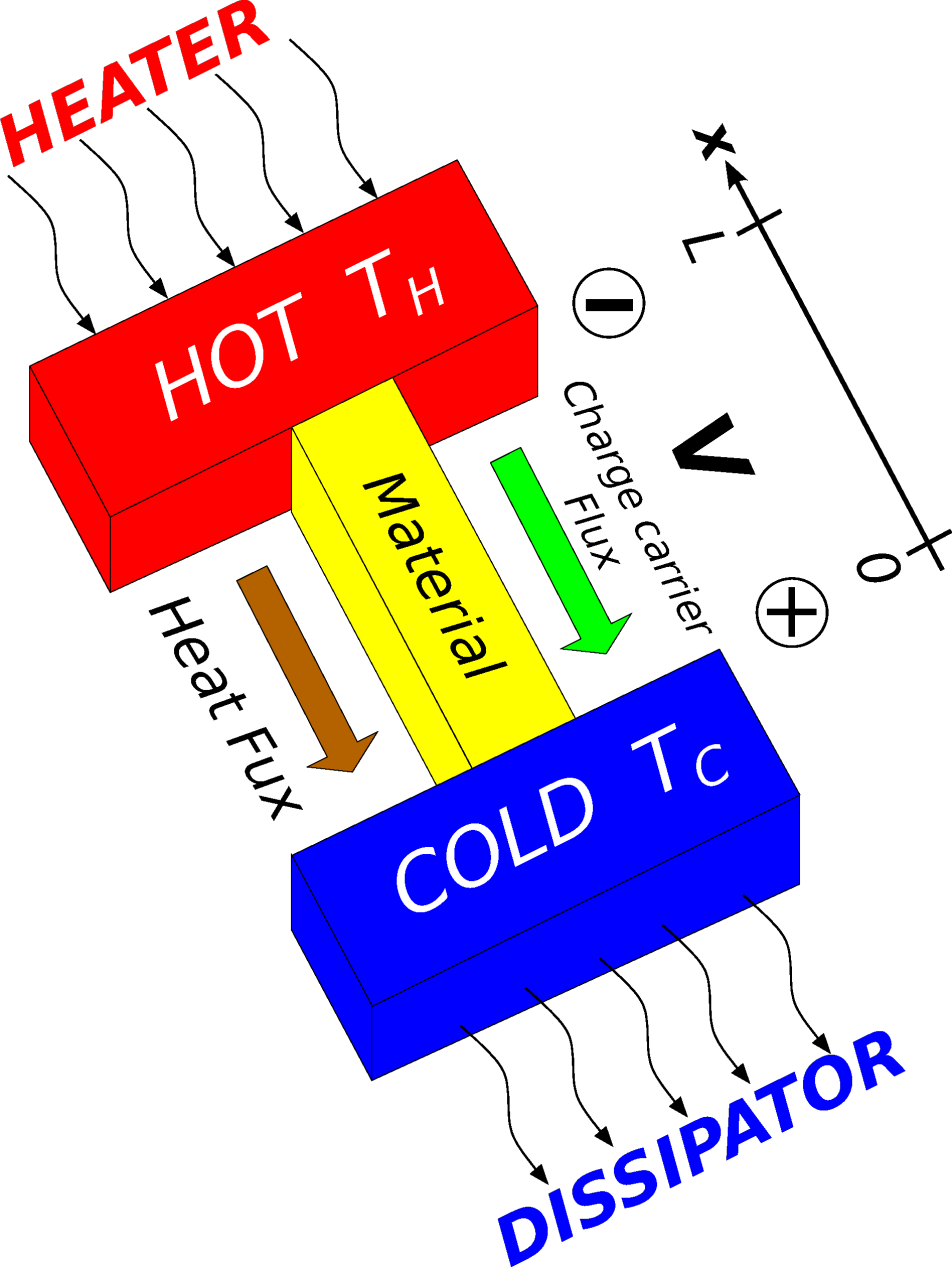
Sketch of a piece of semiconducting material, placed between a hot source (heater, temperature *T*_H_) and a cold source (heat sink, temperature *T*_C_). A potential difference *V* is established between the extremities, due to the charge carrier flux driven by the temperature gradient.

A temperature gradient can be obtained placing a piece of conducting, or semiconducting, material between a hot (temperature *T*_H_) and a cold (temperature *T*_C_) heat source. As an effect of this temperature gradient, both heat and charge carriers (electrons or holes) tend to diffuse through the conductor, from the hot to the cold source. As a consequence of the charge carrier diffusion, a potential difference *V* is established between the hot and the cold extremity of the material (see Figure 1). This effect has been noted for the first time by Thomas Seebeck (1770–1831), who measured a current in a circuit made of different materials and subjected to a temperature gradient. The Seebeck coefficient *S*, also indicated as thermopower, can be written as:

[1]
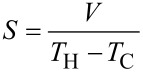


A precise expression for *S* takes into account the temperature gradient *∂T*/*∂x* and the generated electric field ε = −*∂V*/*∂x* at the electrical equilibrium (current density *J* = 0):

[2]
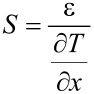


Following this definition, *S* is positive for p-doped semiconductors because if *∂T*/*∂x* > 0 (the temperature increases with *x*), holes tend to diffuse toward negative coordinates. Therefore, at the electrical equilibrium (*J* = 0) ε is positive (see Figure 1, in which a piece of *p* doped semiconductor is represented). In n-doped semiconductors, electrons diffuse from the hot part to the cold part, as do the holes, but their charge sign is opposite, so that *S* is negative.

**Figure 2:**
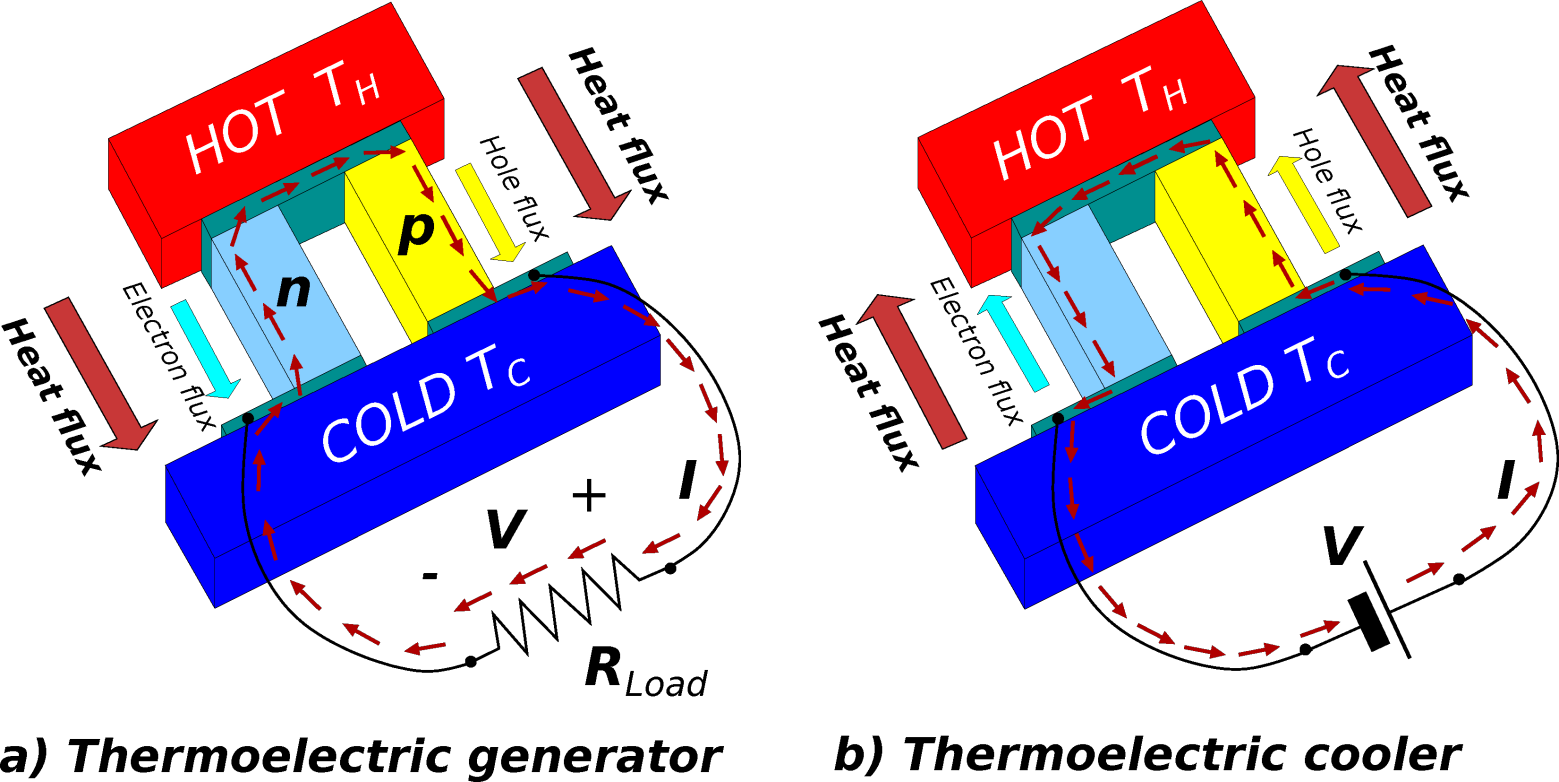
a) Sketch of a thermoelectric generator: two pieces of semiconducting materials with different (opposite) Seebeck coefficient can exploit a temperature gradient to deliver electrical power to an external load *R*_Load_, indicated with *R*_L_ in the text. b) Sketch of a thermoelectric cooler: a battery imposes a current through the legs, so that the flux of the charge carriers brings heat from the cold extremity to the hot extremity of each leg.

Following Kirchhoff’s law, at least two materials with different Seebeck coefficients must be used in order to obtain a potential drop in a circuit in which a temperature gradient is maintained. The basic cell of a thermoelectric generator is made of two semiconducting legs with opposite doping, placed thermally in parallel and electrically in series as schematically shown in Figure 2. Both the heat flux and the charge carrier flux have the same direction, i.e., from *T*_H_ to *T*_C_. The electrical series of the legs, that have opposite Seebeck coefficients (*S*_p_ > 0, *S*_n_ < 0), results in a potential drop proportional to the temperature difference:

[3]
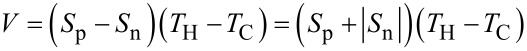


An eventual load, applied to this generator (schematically represented by a resistor *R*_L_ in Figure 2a), can benefit of the carrier flux (electrical current) induced and sustained by the temperature gradient.

Conversely, if an electrical current is forced through the legs by means of an external generator (Figure 2b), heat is transferred from the cold side to the hot side by the charge carrier flux, and the system acts as a cooler. This effect has been investigated for the first time by Jean C. A. Peltier in 1834, who established a relationship between the heat flux Φ and the charge carrier flux (electrical current density *J*) imposed by the external generator:

[4]
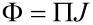


Where Π is the so-called Peltier coefficient. The Seebeck effect and Peltier effect have the same physical basis (the one is the reverse of the other), and an important relationship is established between *S* and Π: Π = *ST*. In principle, the same basic element made of two legs (electrically in series and thermally in parallel) can be used both to generate electrical power, if a temperature difference is maintained (by using for example a heater for *T*_H_ and a heat sink for *T*_C_), and as a cooler, if electrical power is supplied by a battery. Even if in this review a particular emphasis on thermoelectric generation will be given, the same principles could be similarly applied to devices for thermoelectric cooling (Peltier cells). In practical applications, a TEG (or a Peltier cooler) is made of several basic elements placed in series (to increase the voltage) and/or in parallel (for high currents).

The properties of the electrical and thermal transports are resumed by two thermoelectric equations:

[5]
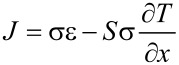


[6]
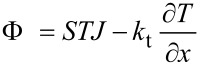


In these equations, the current density *J* is related to the electric field *ε* and to the field generated by the temperature gradient *S∂T*/*∂x*, because of the electrical conductivity of the material, σ. The heat flux Φ depends on the current density *J*, through the Peltier coefficient Π = *ST*, and on the temperature gradient *∂T*/*∂x*, because of the thermal conductivity of the material, *k*_t_. A thermoelectric generator is characterized by its conversion efficiency *η*, defined as the electrical power delivered to the load *R*_L_ divided by the thermal power extracted from the hot source. In terms of heat flux, assuming that the legs are adiabatic on the lateral sides, the heat power exchanged with the hot source per surface unit is Φ_H_ = Φ(*x* = 0). The heat drained by the cold source, and wasted, is Φ_C_ = Φ(*x* = *L*). Therefore, the converted electrical power, delivered to the load, is Φ_H_ – Φ_C_ multiplied by the total cross section surface of the legs, so that the efficiency is:

[7]
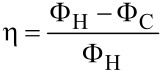


without loss of generality, legs with constant cross section surface have been hypothesized for the sake of simplicity.

Given *T*_H_ and *T*_C_, the total current *I* in the generator depends on the thermoelectric parameters (*S*, σ and *k*_t_), on the geometrical parameters (length and cross section surface of the generator legs), and on the load resistance *R*_L_. Combining the thermoelectricity Equation 5 and Equation 6 with the stationary heat equation *∂*Φ/*∂x* = *εJ*, it is possible to determine *T*(*x*) in the legs, and also Φ_H_ and Φ_C_, so that an expression for the efficiency *η* can be obtained:

[8]
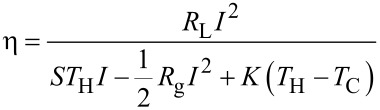


where *R*_g_ and *K* are the total electrical resistance and the total thermal conductance of the generator, which can be evaluated respectively from the material electrical resistivity *ρ* = 1/σ and from the material thermal conductivity *k*_t_, to be combined with the geometrical parameters of the legs (length and cross-section surface). Given a temperature difference *T*_H_ − *T*_C_, the generator acts as a voltage generator with an open circuit voltage *V*_g_ = *S*_total_(*T*_H_ − *T*_C_), where *S*_total_ = *S*_p_ + | *S*_n_ |, and a series (parasitic) resistance *R*_G_. The current *I* depends on the generator electrical resistance *R*_g_ and on the load resistance *R*_L_. In the evaluation of these expressions, the variation with temperature of the thermoelectric parameters *S*, σ and *k*_t_, has been neglected. These parameters need to be evaluated at the average temperature 
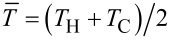
. In order to obtain the maximum conversion efficiency, the load resistance *R*_L_, which appears both explicitly an through the current *I* in Equation 8, needs to be optimized. By simple mathematical passages, it is possible to show that the optimum load condition, for which *η* is maximum, is:

[9]
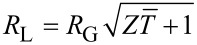


where the factor *Z*:

[10]
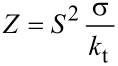


is the parameter that qualifies the materials for thermoelectric conversion. If optimum load conditions are established, the maximum conversion efficiency is:

[11]
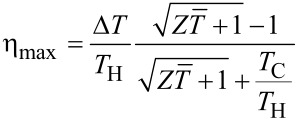


The first factor of this expression, Δ*T*/*T*_H_, is the Carnot efficiency, that is the maximum conversion efficiency fixed by the second principle of thermodynamics. The second factor depends on the parameter *Z*, or better on the dimensionless parameter 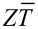, that must be as high as possible to have high efficiencies. If 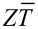 → ∞, the efficiency *η* has the maximum value obtainable with the two heat sources *T*_H_ and *T*_C_: *η* = Δ*T*/*T*_H_, i.e., the Carnot limit. The development of a good thermoelectric material should aim to obtain a factor *Z* = *S*^2^σ/*k*_t_ as high as possible: A good thermoelectric material should have high Seebeck coefficient *S* and electrical conductivity σ, and small thermal conductivity *k*_t_.

### Materials for thermoelectricity

Several experimental works on thermoelectric materials are devoted to maximize the power factor *S*^2^σ, which is proportional to the power delivered to the load *R*_L_. Given two heat sources (or better a heat source *T*_H_ and a heat sink *T*_C_), the optimization of *S*^2^σ leads to the maximization of the power that a TEG can deliver to the load. In this respect, graphene [11] could offer interesting opportunities, in particular for its high electrical conductivity σ. The Seebeck coefficient of pristine graphene is of the order of 10–100 μV/K [12–13]. Several solutions for increasing this value up to several hundreds of μV/K, such as plasma etching treatments [11], have been carried out successfully. A great enhancement of *S* has been predicted in graphene nanoribbons [14–15], and the use of a suitable array of nanoelectrodes has been proposed for obtaining a giant thermoelectric power [16].

The maximization of the power factor *S*^2^σ is important in those applications that require a power as high as possible, and that have enough thermal energy available on the hot source. However, in many practical applications, one of the key points is to exploit as much as possible the thermal energy that is available on the heat source, so that energy conversion efficiency, more than maximum delivered power, is the main issue to be faced. In other words, for the majority of applications the main target is to optimize energy, instead of maximize power. As for example, the application of TEGs as alternative to photovoltaic cells must aim for the maximum exploitation of solar energy, hence to the maximum conversion efficiency. A strong increase of conversion efficiency would also allow for the minimization of the heat dissipated on the cold side, so that the exchanging surface of the dissipating system can be reduced.

Therefore, the development of a good thermoelectric material, useful for the majority of thermoelectric applications, should aim to maximize the efficiency *η* (see Equation 11). Hence, the figure of merit 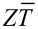, and in particular the factor *Z* = *S*^2^σ/*k*_t_, must be as high as possible. This means that a good thermoelectric material, as previously stated, should have a high Seebeck coefficient *S* and a high electrical conductivity σ, but also a low thermal conductivity *k*_t_. In this respect, graphene exhibits a very high thermal conductivity, higher than 3000 W/mK [17], so that the figure of merit of graphene-based thermoelectric generators would be very small.

**Figure 3:**
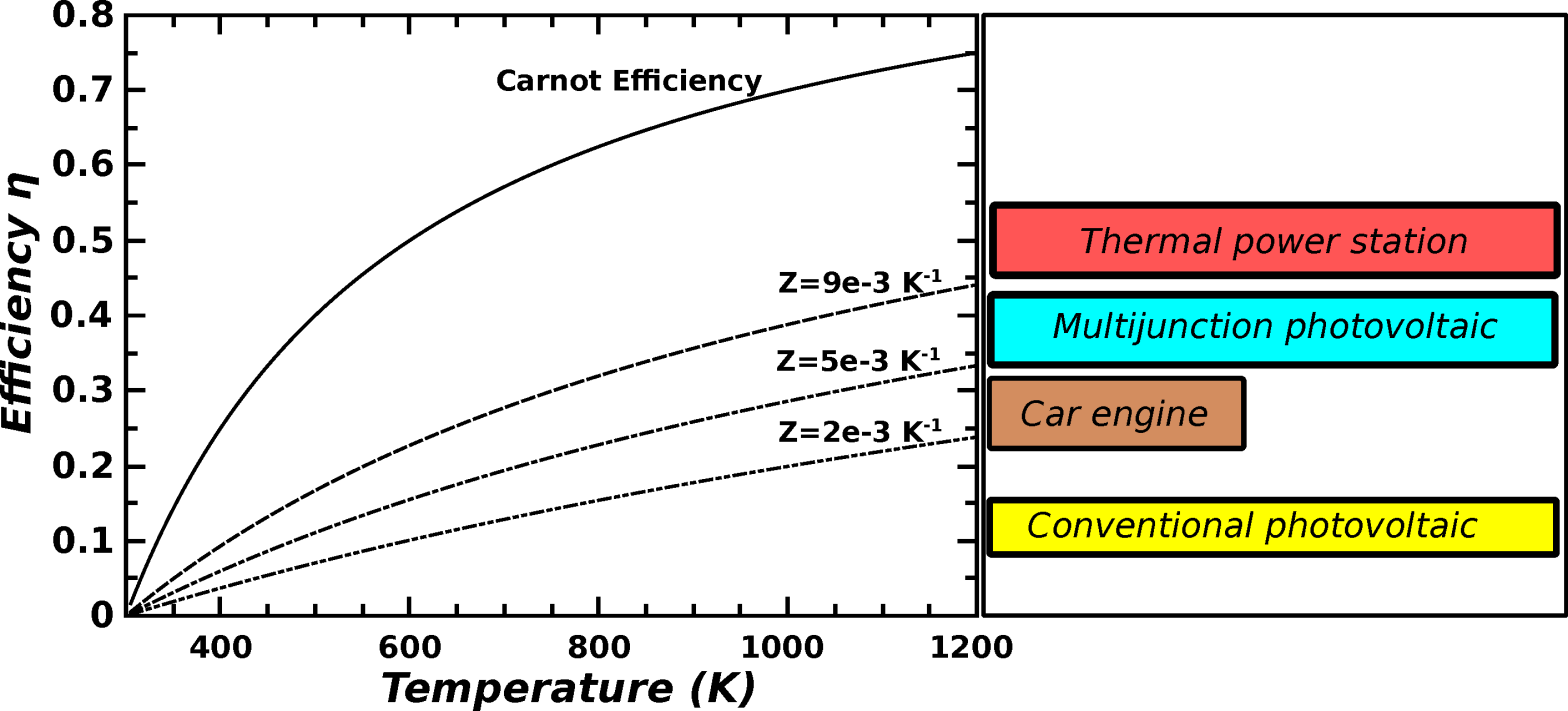
Efficiency of a thermoelectric generator for different values of the material-dependent parameter *Z* = *S*^2^σ/*k*_t_, as a function of the hot source temperature *T*_H_. The cold source temperature is *T*_C_ = 300 K. The efficiency of conventional techniques for energy production is also reported for comparison.

In Figure 3 the efficiency of a thermoelectric generator is reported as a function of the hot source temperature *T*_H_. The curves have been evaluated with Equation 11, assuming that the cold source is maintained at room temperature (*T*_C_ = 300 K). Curves for different values of the material-dependent parameter *Z* are shown. The maximum efficiency theoretically obtainable (Carnot efficiency Δ*T*/*T*_H_) is also reported as a function of *T*_H_. In the figure, TEG efficiencies are also compared with a few conventional techniques for energy production (see the sketch on the right). It can be seen that if research can provide materials with sufficiently high values of the parameter *Z*, thermoelectric generation will become comparable with other techniques. It must be noted that the parameters *S*, σ and *k*_t_, and thus the parameter *Z*, are strongly temperature dependent. Furthermore, these parameters depend on the temperature through non-linear relationships, that in general can be determined only by experimental measurements. Therefore, a numerical solution of the thermoelectric equations (Equation 5 and Equation 6), which takes in account the dependence of the material parameters on the temperature, must be implemented for an exact calculation of the TEG efficiency [18]. The graphs reported in Figure 3 have been evaluated considering *Z* constant in the temperature range from *T*_H_ to *T*_C_. Hence, these curves can be used only for a rough indication of the *Z* value that is required for a desired efficiency. The *Z* values suggested by the graph can be interpreted as the optimum *Z* parameter that should be maintained in the temperature range between the selected *T*_H_ and 300 K.

In order to compare the thermoelectric performances, in previous works on thermoelectricity the so-called figure of merit 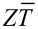 (in general *ZT* ) has been introduced as a comparison parameter. *ZT* is a dimensionless parameter that appears explicitly in the efficiency expression (Equation 11), and a material with a *ZT* value greater than 1 is, in general, considered acceptable for thermoelectric applications. However, the use of the figure of merit *ZT* as performance parameter can be misleading, because it is not directly connected to the efficiency that strongly depends on the temperature difference. Furthermore, the temperature dependence of *ZT* is even stronger than that of the *Z* parameter. If for example a material shows *ZT* = 2.0 at an average temperature of 400 K, this means that *Z* must have an almost constant value of 5 × 10^−3^ K^−1^ in the temperature range between 300 and 500 K (assuming the room temperature of 300 K as cold source temperature *T*_C_). For TEGs that must work with small temperature differences at about room temperature, as for example in cooling/generating systems for domestic plants, a material with 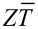 = 2.0 can be thought to be a very good one because *Z* = 7 × 10^−3^. However, the temperature difference is very small, hence the the expected efficiency will be very low. For these reasons, in the following sections the *Z* parameter will be considered for comparing TE materials.

**Figure 4:**
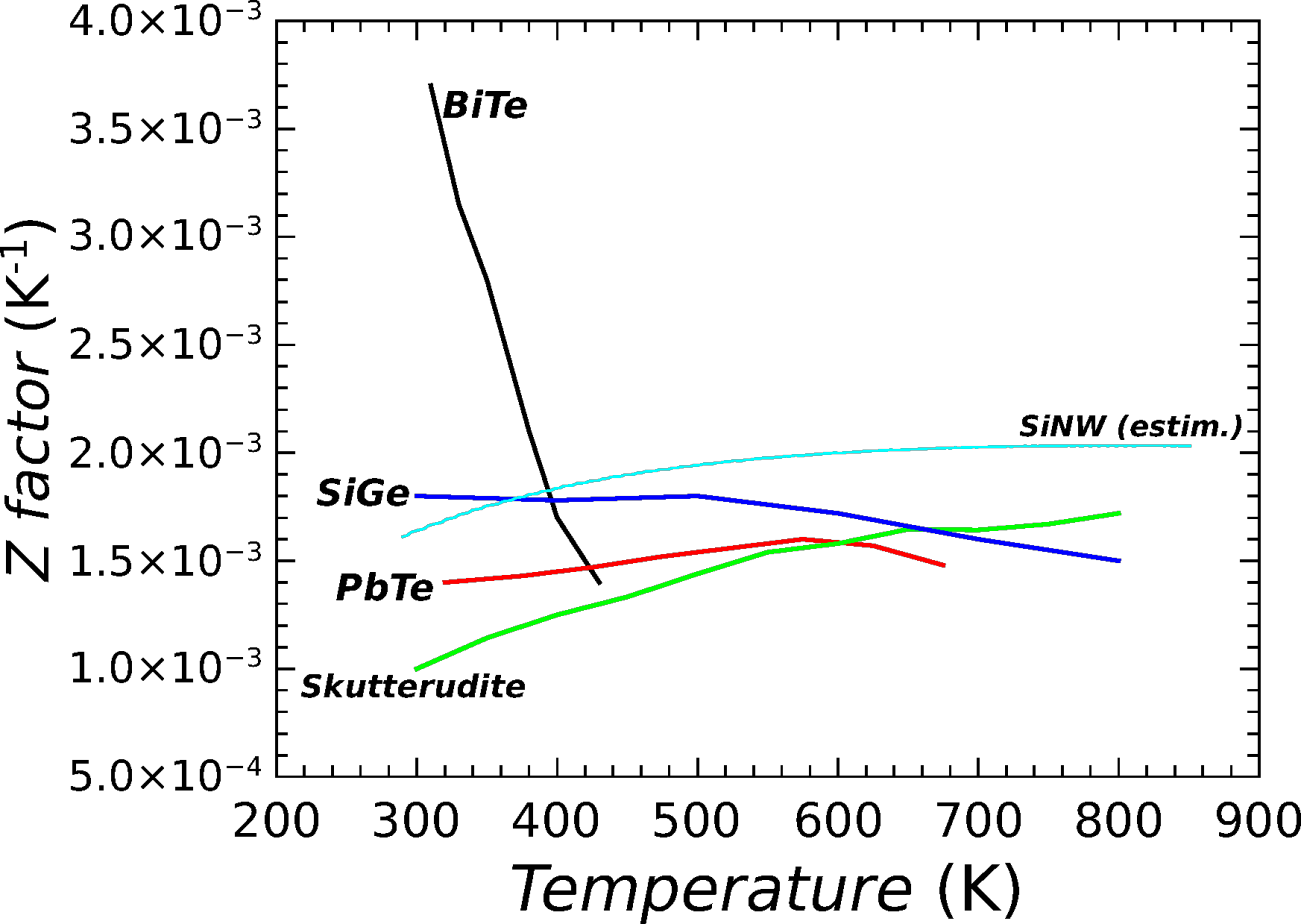
The *Z* parameter is reported as a function of temperature for few most common TE materials (n type). Experimental data from literature have been used, and in particular: curve BiTeSe from [23]; PbTe from [28]; skutterudites from [37]; and SiGe from [38]. Numerical estimation of the *Z* parameter for a silicon nanowire 50 nm wide and n-doped 5 × 10^19^ cm^−3^ is also reported.

Many tellurium compounds showed interesting TE potentialities. The most widely used material for thermoelectricity, extensively studied since 1954 [19–21], is Bi_2_Te_3_. It is a narrow gap (about 160 mV at 300 K) semiconductor, whose electrical conductivity can be increased by doping, either n- or p-type. Its moderately high Seebeck coefficient (between 0.1 and 0.25 mV/K), and in particular its low thermal conductivity (between 2 and 3 W/mk at room temperature), gives a *Z* factor of about 2.5 × 10^−3^ K^−1^ at room temperature (*ZT* of the order of 0.7–0.8). However, the *Z* factor reaches its maximum value in a narrow temperature range (about 50 K wide) around 300 K. Hence, TEGs based on Bi_2_Te_3_ can be usefully employed only for small temperature differences at about room temperature. Higher *Z* factor values can be obtained by ternary alloys based on bismuth/tellurium and antimony (typically p-doped) or selenium (typically n-doped) [22–25]. Values of the *Z* factor in excess of 4.5 × 10^−3^ K^−1^ (*ZT* in excess of 1.4) at room temperature have been found. A further increase of the *Z* factor has been measured in nanostructured bismuth antimony telluride alloys, because phonon scattering at nanocrystal boundaries gives a reduced thermal conductivity [26]. Hovewer, TEGs based on bismuth telluride compounds have a small operating temperature range, because the *Z* factor rapidly decreases well below 2 × 10^−3^ K^−1^ for *T* > 400 K, as shown in Figure 4. Lead telluride (PbTe) compounds [27–28] exhibited a smaller *Z* value, of the order of 1.5 × 10^−3^ K^−1^, but its value is almost stable on a large temperature range (from room temperature up to 800 K, see Figure 4). Therefore, PbTe-based TEGs can operate with large temperature differences, so that they can pursue higher efficiencies with respect to other tellurium based materials. Nanocrystalline PbTe compounds [29–30] showed an increment of the *Z* factor in excess of 2 × 10^−3^ K^−1^, still maintaining a large temperature range. Even if all these TE characteristics (high *Z* factor and large temperature range) of tellurium compounds are very attractive for applications, it must be mentioned that tellurium is a very rare material on the surface of Earth. Moreover, tellurium is a toxic material, thus disposal of tellurium-based TEG devices would arise noticeable environmental problems. For these reasons, research on thermoelectricity is focussing on tellurium-free materials. Skutterudites (CoSb_3_), filled with rare or alkaline earths or metals, show interesting perspectives because of their reduced thermal conductivity [31]. Slack suggested [32] that the random distribution of filling ions as well as their “rattling” effect increases the phonon scattering on a large spectrum, so that the thermal conductivity is strongly reduced and *Z* is quite high. Several filling elements, such as La [32], Co [33], Ta [34], and others, have been experimented. Recently, filled [35–37] skutterudites were found with *Z* factors of about 2 × 10^−3^ K^−1^, for temperatures up to 800 K. Skutterudites are, in general, based on elements that are not very rare, however sustainability problems could arise from the presence of cobalt.

A big research effort has been devoted to the enhancement of TE properties of compounds based on silicon. Silicon is one of the most well known materials both from the physical and from the chemical point of view, it is abundant on the surface of Earth, and it is very sustainable. Furthermore, devices based on silicon could exploit a large operating temperature range, because silicon is a very stable material for temperatures in excess of 900 K. Silicon–germanium alloys, SiGe [38–39], and superlattices [40–41] showed a good *Z* factor value, of the order of 2 × 10^−3^ K^−1^ at 800 K. Furthermore, they can be used for power generation in devices exploiting temperature differences between 900 K and 300 K. Other silicon compounds are actually under development. Very promising results have been obtained with magnesium silicide (Mg_2_Si) [42–43] compounds, which are low cost and non-toxic. Highly boron-doped nanocrystalline silicon [44] is another promising material for its increased electrical conductivity and Seebeck coefficient compared to bulk silicon.

### Advantages of nanostructured materials for thermoelectricity

**Figure 5:**
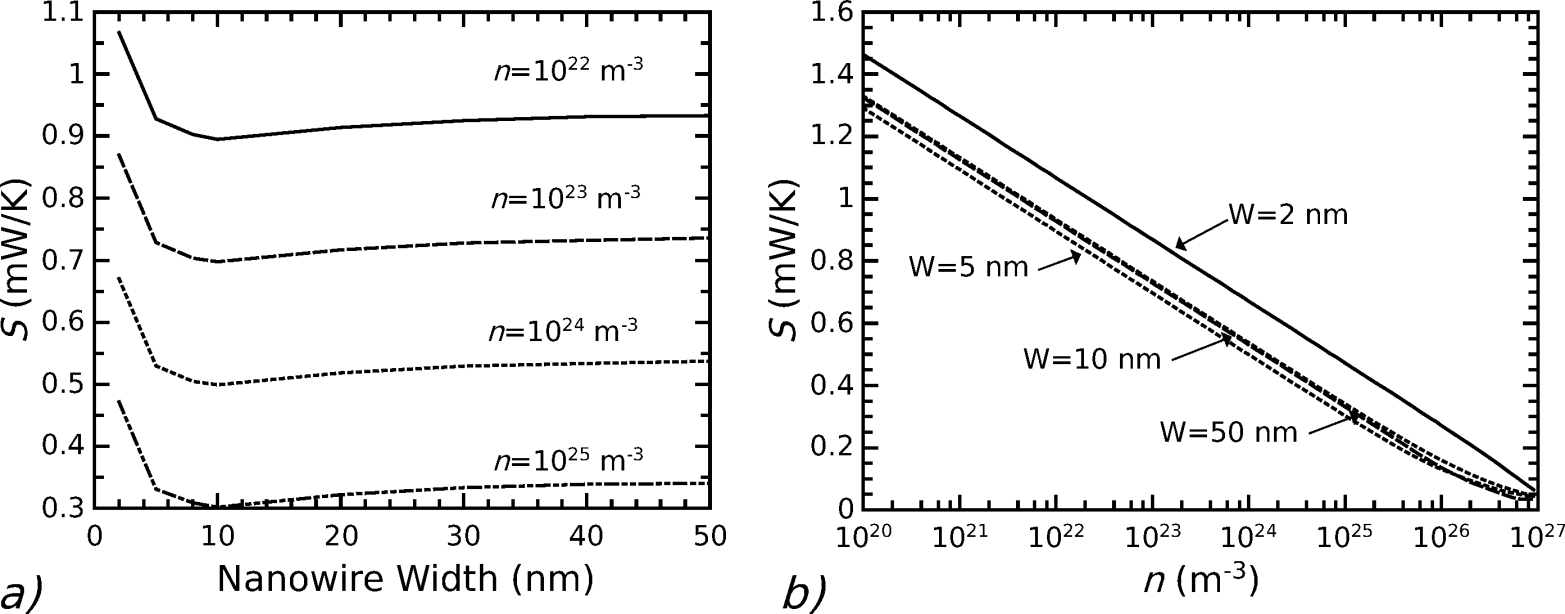
Panel (**a**): Seebeck coefficient in silicon nanowires with triangular cross section as a function of the nanowire width, for different values of n-doping. Panel (**b**): Seebeck coefficient in silicon nanowires as a function of the doping for different nanowire widths. Reproduced with permission from [18], Copyright (2013) AIP publishing LLC.

Thermoelectric parameters are enhanced in structures with reduced dimensionality. As pointed out by Dresselhaus and co-workers in pioneering theoretical works [45–46], and further investigated also by other authors [18,47–48], the Seebeck coefficient increases in bidimensional, or monodimensional (nanowires), structures. The Seebeck coefficient strongly depends on the distribution in energy of the charge carriers (electrons or holes), and in particular it increases when the average difference between the carrier energies and the Fermi energy increases. In low dimensional systems, the density of states is reshaped with respect to bulk systems, in such a way that charge carriers are spread to higher energies. This produces an increase not only of *S* but also, in principle, of the electrical conductivity σ, with a benefit for the *Z* = *S*^2^σ/*k*_t_ parameter. However, first of all structures with diameters of the order of a few nanometers are requested in order to obtain a noticeable increase of the Seebeck coefficient with respect to its bulk value. Moreover, the electrical conductivity of very small nanostructures is strongly reduced by the surface scattering of charge carriers. As an example, Figure 5 shows the Seebeck coefficient evaluated for silicon nanowires with triangular cross section [18], as a function of the nanowire width (triangular base) *W*: *S* increases only if the nanowire width is reduced below 5 nm. It is to be noted that *S* increases noticeably for low doping values, because the Fermi energy level decreases with respect to the bottom of the conduction band, and consequently the energy difference between charge carriers and Fermi level increases: Values of the order of 1 mV/K can be obtained in silicon for doping values smaller than 10^16^ cm^−3^.

The main point in the use of nanostructured materials for thermoelectricity is the strong reduction of the thermal conductivity *k*_t_ compared to the bulk value. This strong reduction of *k*_t_ has been measured in experimental works on nanowires [8,49–50], and confirmed by several theoretical studies [51–54]. The thermal conductivity of the material *k*_t_ can be written as the sum of two main contributions, *k*_t_ = *k*_e_ + *k*_ph_, hence the parameter *Z* can be written as *Z* = *S*^2^σ/(*k*_e_ + *k*_ph_). The first term *k*_e_ takes into account the heat brought by the charge carriers (electrons or holes) that diffuse from the hot part *T*_H_ to the cold part *T*_C_. The second term *k*_ph_ takes into account the heat conduction through the material crystalline lattice, due to phonon propagation. *k*_e_ (*e* for electrons, the same for holes) depends on charge carrier concentration and mobility, and it is strictly connected with the electrical conductivity σ. The thermal conductivity of metals is principally due to *k*_e_, which is related to the electrical conductivity σ through the well-known Wiedemann–Franz law:

[12]
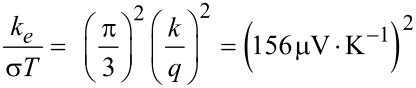


The phonon contribution to the thermal conductivity, *k*_ph_, is instead predominant in semiconductors. For example, in bulk silicon *k*_t_ = 148 W/mK, meanwhile *k*_e_, which depends on doping (i.e., on the electron or hole concentration), is always smaller than 1 W/mK for doping values up to 10^19^ cm^−3^. When the dimensions of nanostructures (as for example the nanowire width/diameter) become comparable with the phonon mean free path, which is of the order of several tens of nanometers at room temperature, the phonon propagation is limited by the surface scattering. It has been observed that the effect of thermal conduction reduction by surface phonon scattering is enhanced in nanostructures with rough surfaces. Several experimental works have been dedicated to the measurement of the thermal conductivity in rough silicon nanowires [9,55–59], and it was found that *k*_t_ decreases down to few W/mK (bulk silicon thermal conductivity = 148 W/mK) in nanowires with diameters of several tens of nanometers (20–100 nm). This value is well below the so-called Casimir limit [60–61], which is the minimum thermal conductivity theoretically obtainable when a completely diffusive phonon scattering on the nanowire surface is assumed. On the basis of these intriguing experimental results, several works [62–67] developed theoretical models for phonon boundary scattering, taking into account coherent effects. For an appropriate scale of surface roughness, these coherent effects can be important for phonon reflection and suppression. Therefore, accordingly with the experimental results, these models confirm that a phonon thermal conductivity below the diffusive limit can be obtained in rough nanowires.

**Figure 6:**
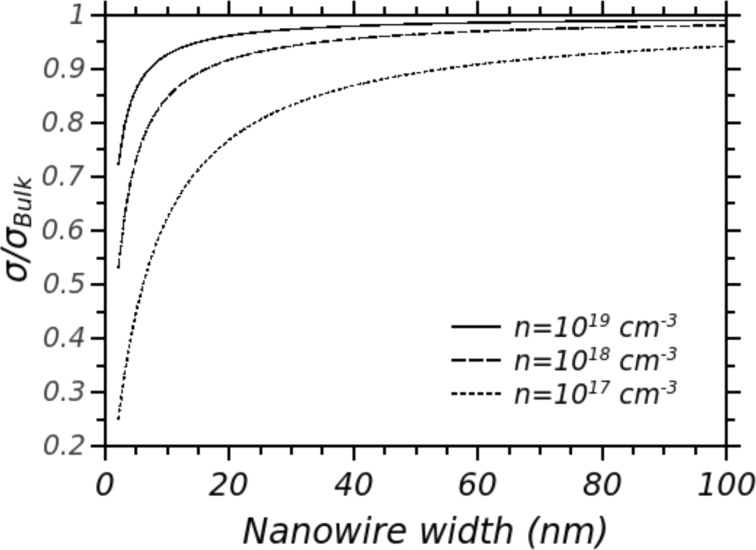
Electrical conductivity σ of a silicon nanowire as a function of the nanowire width (triangular cross section), normalized with respect to the bulk electrical conductivity σ_Bulk_.

On the other hand, the electrical conductivity in rough nanowires is only slightly affected by the surface scattering of charge carriers. Figure 6 shows the electrical conductivity σ in n-doped silicon nanowires, normalized with respect to the value σ_Bulk_ obtained in bulk silicon with the same doping concentration. These curves have been numerically evaluated with a simple model that takes into account a completely diffusive scattering of electrons on the nanowire surfaces. Variations of the electrical conductivity, with respect to its bulk value, are significant only in nanowires narrower than 20 nm. This is especially true in particular for high doping values, for which electron mobility is limited by impurity scattering and the electron mean free path is very small with respect to the nanowire diameter. Thus, in nanowires wider than 20–40 nm, phonon thermal conduction *k*_ph_ can be strongly reduced by increasing the surface roughness, meanwhile the electrical conductivity remains comparable with that of bulk silicon. Therefore, a strong enhancement of the factor *Z* = *S*^2^σ/(*k*_e_ + *k*_ph_) can be obtained in rough nanowires wider than 20–40 nm. If *k*_ph_ is reduced to values smaller than *k*_e_, doping can be optimized so that the *Z* factor can be further increased [18]. If *k*_ph_ < *k*_e_, the *Z* factor becomes *Z* = *S*^2^σ/(*k*_e_ + *k*_ph_) ≈ *S*^2^σ/*k*_e_ and, as mentioned above, the Wiedemann–Franz law states that the ratio σ/*k*_e_ is constant. Thus, a suitable decreasing of the doping leaves this value unchanged, while the *Z* factor improves because *S*, which appears squared in the *Z* expression, increases. In Figure 4 the estimated *Z* factor for a silicon nanowire 50 nm wide, n-doped 5 × 10^19^ cm^−3^, is reported for a comparison with other TE materials. An optimistic value of *k*_ph_ = 0.8 W/mK (*k*_e_ given by the doping) has been assumed for the numerical estimation of *Z*.

### Nanostructured silicon for thermoelectricity

In the last years, nanowires have been extensively investigated for their potentialities as advanced electronic devices [68–70] and as sensing elements [71–73]. For these purposes, devices based on single, or very few, silicon nanowires have been fabricated exploiting both bottom-up and top-down approaches. However, devices for thermoelectric generation must be able to handle high powers, that means high currents and voltages. Thus, techniques for the massive and reliable production of a huge number of nanowires, well organized and electrically interconnected in a well determined way, need to be developed. Bottom-up approaches are based on the crystalline growth of nanowires by means of chemical vapour deposition (CVD) techniques. The most common CVD technique for the fabrication of silicon nanowires is the vapor–liquid–solid (VLS) growth [74–75], developed in particular by the group of Lieber at Harvard [76–80], and rapidly diffused and applied by several other groups [81–85]. The VLS growth is based on the catalytic effect of metal (gold or iron) nanoparticles, deposited on a silicon substrate. At high temperatures, an eutectic alloy is formed among metal and Si, supplied by a silane (SiH_4_) flux (or others silicon-based gases). A precise calibration of silane flux and reaction temperature gives a supersaturation of the melt and induces a transformation from the liquid alloy phase to solid Si that crystallizes under the metal nanoparticle. In this way, crystalline silicon nanowhiskers grow perpendicularly to the substrate.

Exploiting the VLS-CVD technique, silicon nanowires have been grown between small suspended silicon masses, fabricated by micromachining techniques applied to silicon-on-insulator (SOI) substrates [86–87]. The silicon masses are maintained at different temperatures, so that a thermoelectric microgeneration has been obtained.

The fabrication of silicon nanostructures [88–89] and nanowires [69,90–96] by means of top-down approaches starts from a macroscopic structure, such as a silicon wafer. Suitable patterns are defined on the wafer surface by advanced lithographic tools, such as electron beam lithography [90–91], atomic force lithography [94–95,97] and even optical lithography [69,93]. Etching, oxidation and other fabrication processes are then used to define structures with nanometric dimensions. Top-down fabrication is somewhat more complex compared to the bottom-up approach that allows for the massive, low-cost, production of nanostructures. However, the top-down fabrication allows for the simultaneous fabrication of nanostructures and nanowires together with contacts, connections and control gates, so that the fabrication process yields fully functional devices. Conversely, bottom-up approaches allow for an easier production of nanowires, but then the fabrication of devices requires the development of complex procedures for the positioning of nanowires with respect to contacts and connections.

**Figure 7:**
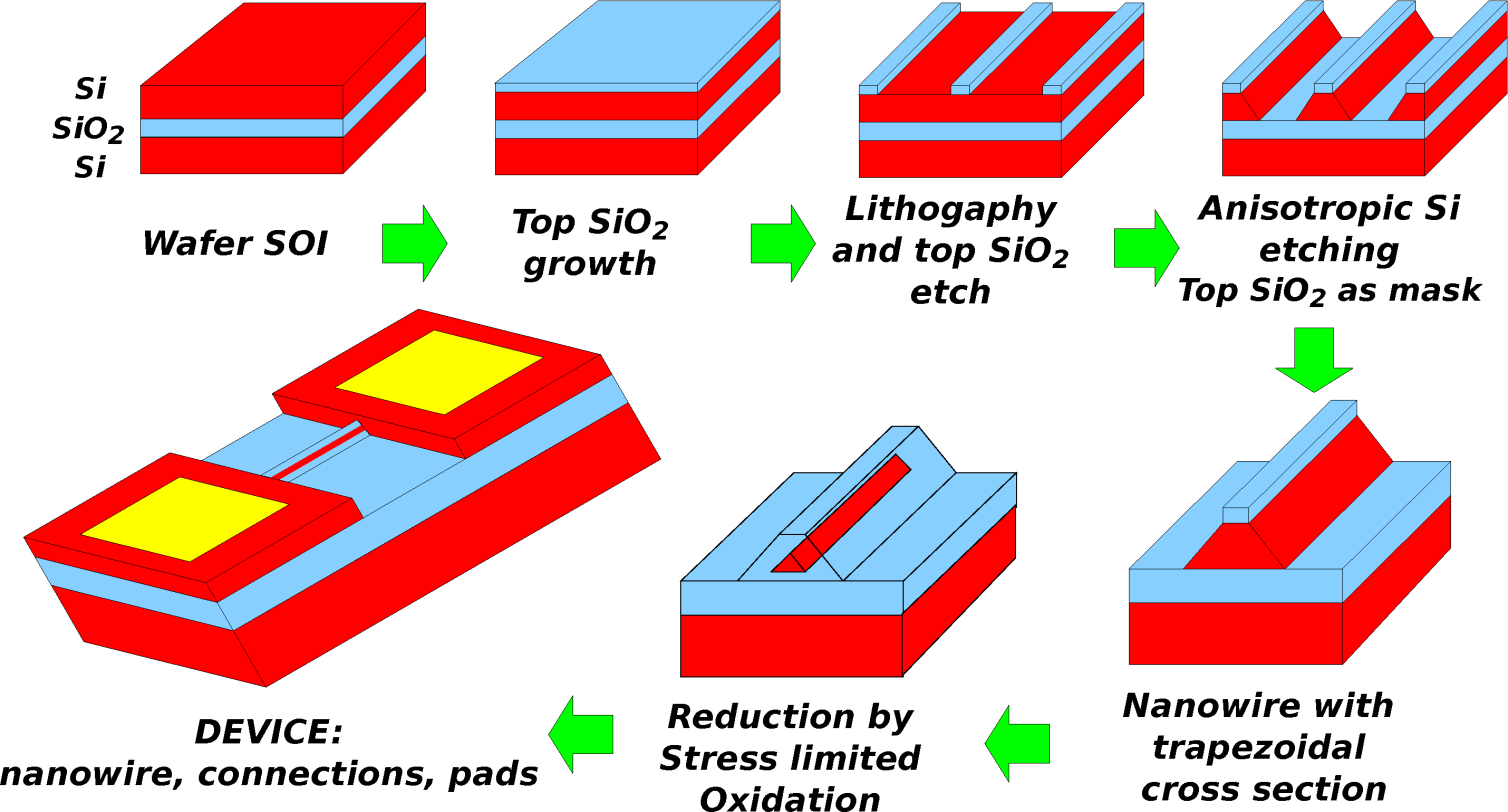
Sketch of a top-down fabrication process for a device based on a silicon nanowire.

A typical top-down process, based on electron beam lithography, anisotropic silicon etching and stress-limited oxidation, is shown in the sketches of Figure 7. This process [91–93] has been developed on a silicon-on-insulator (SOI) substrate, <100> oriented, which is becoming largely employed in the semiconductor industry for the production of integrated circuits. The same process can be used on substrates with different thicknesses of the silicon top layer, in the range between 100 and 300 nm, by adapting the mask dimensions and oxidation parameters. The doping of the silicon top layer can be tailored, both as a type (n or p) and as doping concentration, by exploiting standard silicon doping processes. A 50–80 nm thick silicon dioxide (SiO_2_) top layer, to be used as a mask for the silicon etching, is grown by dry thermal oxidation. This top SiO_2_ layer is patterned by means of electron beam lithography through standard poly(methyl methacrylate) (PMMA) resist. An etching with well calibrated buffered HF (BHF) allows for the transferring of the pattern from the PMMA to the SiO_2_ layer (reactive ion etching can be used as alternative). Once the SiO_2_ mask is defined, the top silicon layer is etched anisotropically. Plasma etching/ reactive ion etching (RIE), which is a standard process in integrated circuit fabrication, can be used. However, a simple and more convenient technique is the wet silicon anisotropic etching in alkaline solutions [98–99], typically based on potassium hydroxide (KOH) or tetramethylammonium hydroxide (TMAH). As for example, the silicon top layer can be etched in KOH 35% in volume at 43°C for 5–7 min: The etching time is not very critical, because the etching stops (it is very low) on the buried oxide layer, and on {111} silicon crystalline planes. At the end of the etching process, the nanowire has a very regular trapezoidal cross-section, the minor base of which at the top is a {100} plane with a width *W*_top_ determined by the lithography. The sloping walls are {111} crystalline planes, where the anisotropic etching stopped. The major base at the bottom has a width *W* that depends on *W*_top_ and on the thickness of the silicon top layer. A trapezoidal cross section allows for an easy and well controllable reduction of the nanowire width by stress-limited oxidation [91–92]. In particular, the silicon oxidation proceeds faster on the sloping sides (that are {111} planes) of the sections, because the oxide growth rate is faster in <111> directions.

**Figure 8:**
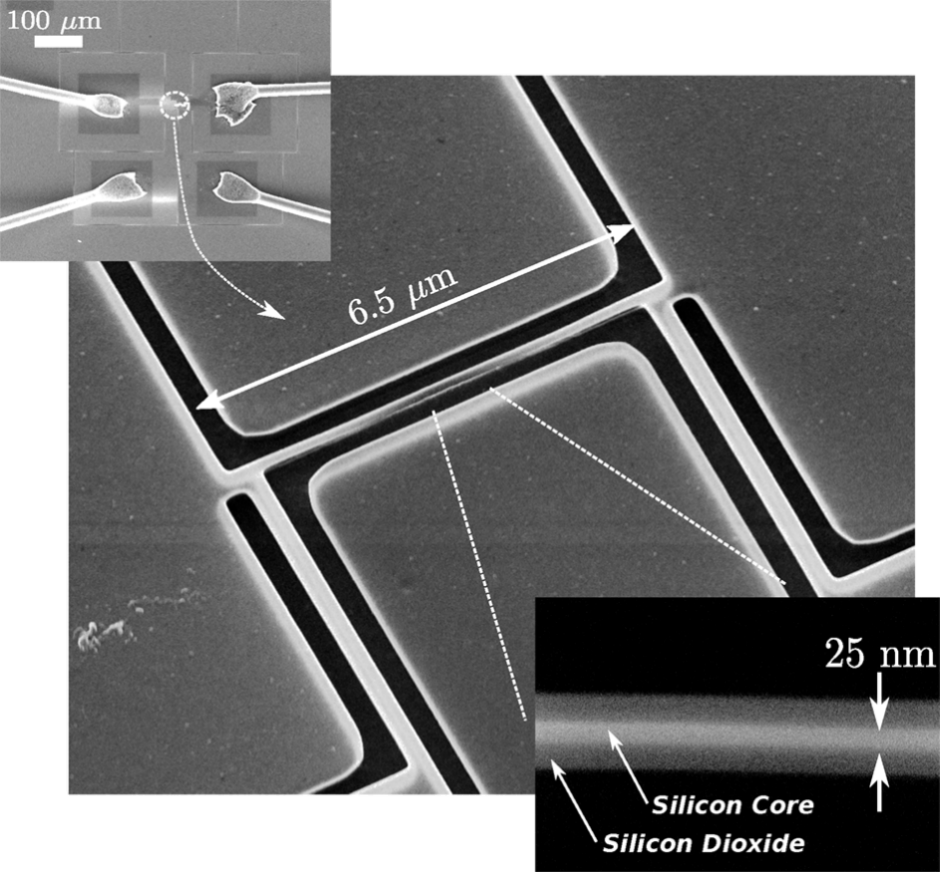
A composition of SEM images of a device, based on a single silicon nanowire positioned between four contacts to be used for electrical characterization. The top-down process allows the simultaneous fabrication of the nanowire and of the interconnections-contacts. Top left: overview of the device, middle: nanowire with two contacts at the extremities and two side contacts, bottom right: enlargement of the central region. For more details refer to the text.

A correct design of the top width *W*_top_, defined by lithography, with respect to the top silicon layer thickness, allows for the formation of a triangular cross section during the oxidation process. Once a triangular cross section is obtained, the increasing of the oxide volume induces a mechanical stress that limits the oxidation rate. Therefore, a well controlled reduction of the nanowire width can be obtained simply by controlling the oxidation temperature and time. With this technique, a very narrow nanowire can be fabricated even if a relaxed lithography, as for example optical lithography, is used for the definition of the initial *W*_top_. Figure 8 is a composition of SEM images of a typical device based on a top-down fabricated silicon nanowire. The top inset is a overview of the device, showing metal pads, bonded with microwires to external connections. The main image shows the nanowire, which is 6.5 μm long. It is fabricated together with two contacts at the extremities and two side contacts to be used for four point measurements. The bottom inset shows an enlargement of the central region: the silicon nanowire core, 25 nm wide, is embedded in silicon dioxide. A calibrated BHF etching, which can be performed at the end of the oxidation process, allows for the tailoring of the silicon dioxide thickness around the nanowire silicon core.

**Figure 9:**
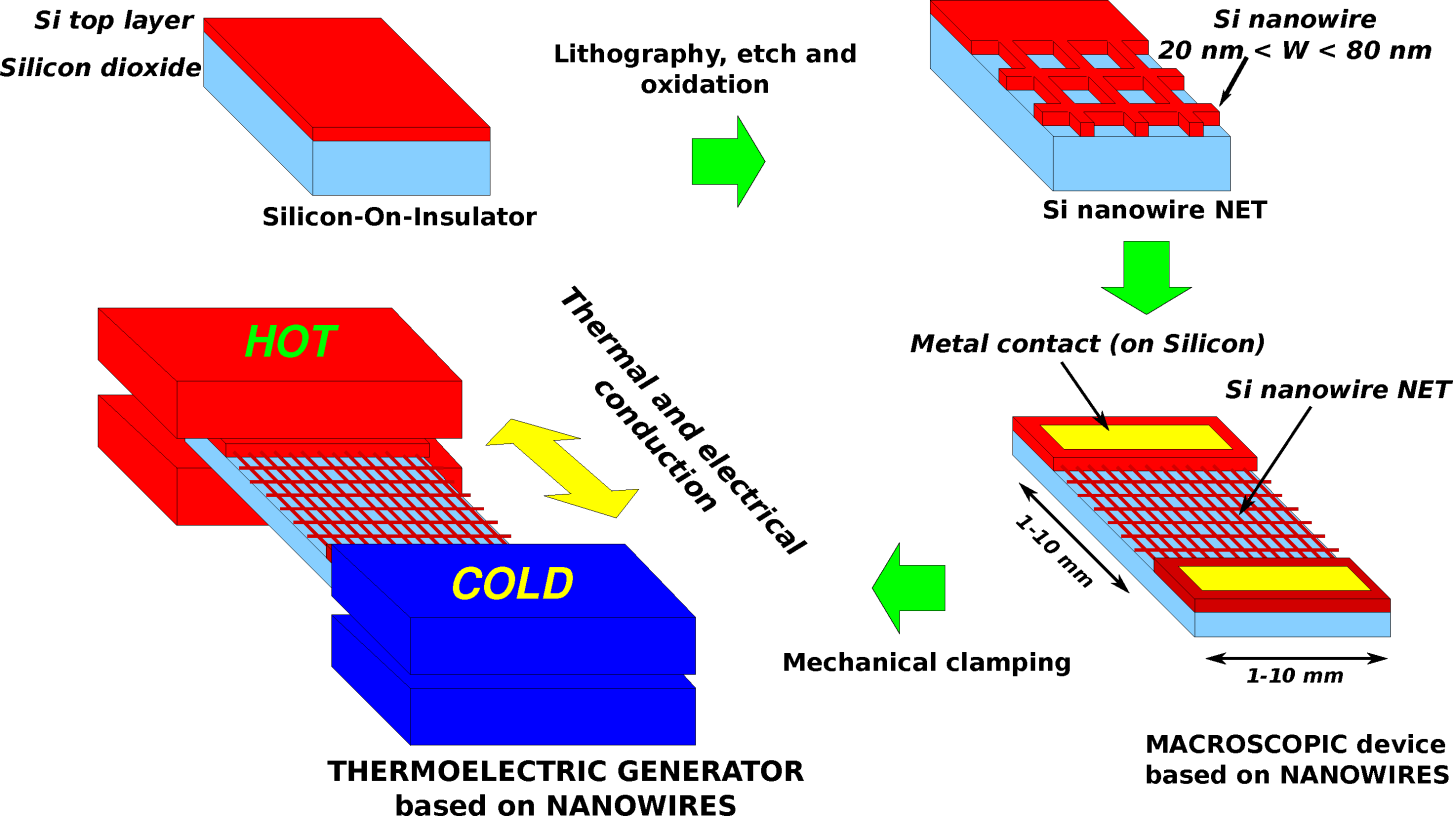
Sketches of the fabrication and mechanical clamping of a macroscopic thermoelectric generator based on nanowires.

**Figure 10:**
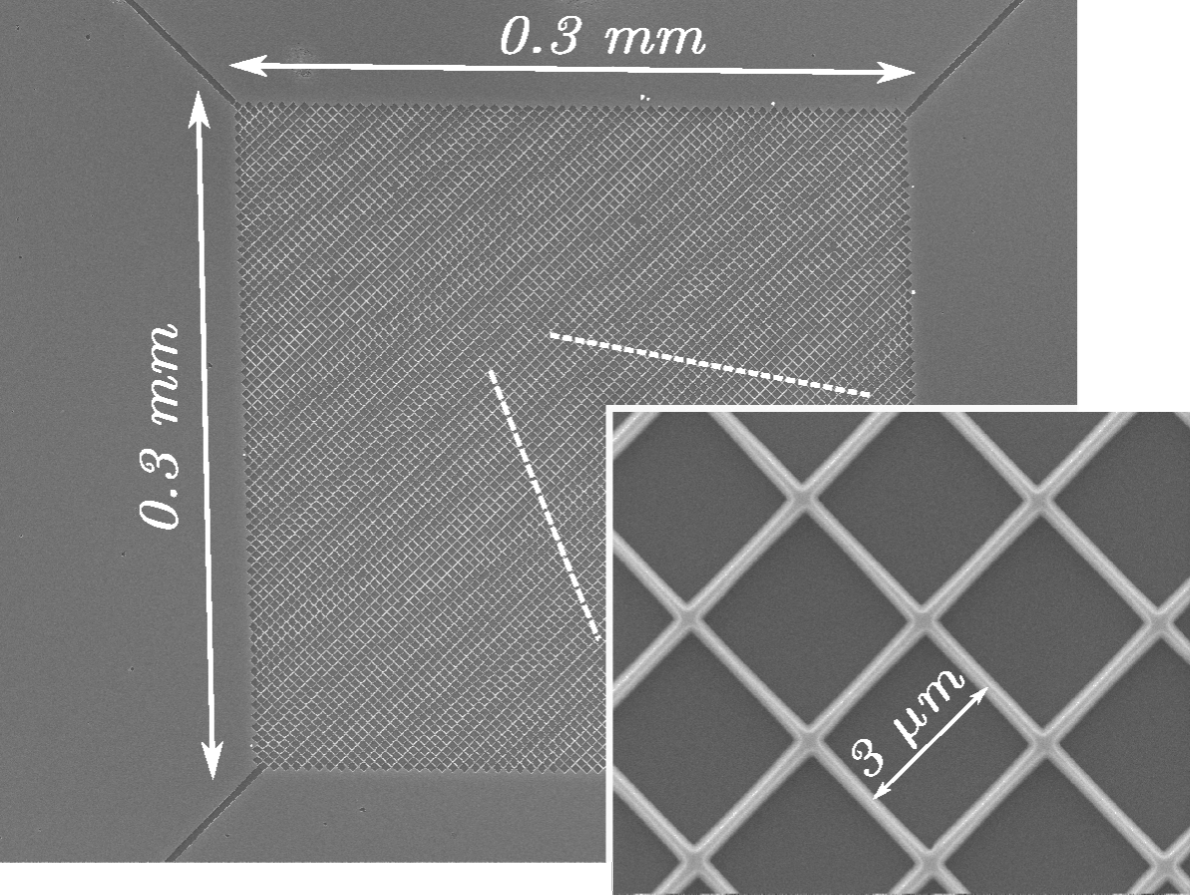
Composition of SEM images of a large area network, made of silicon nanowires 3 μm long.

**Figure 11:**
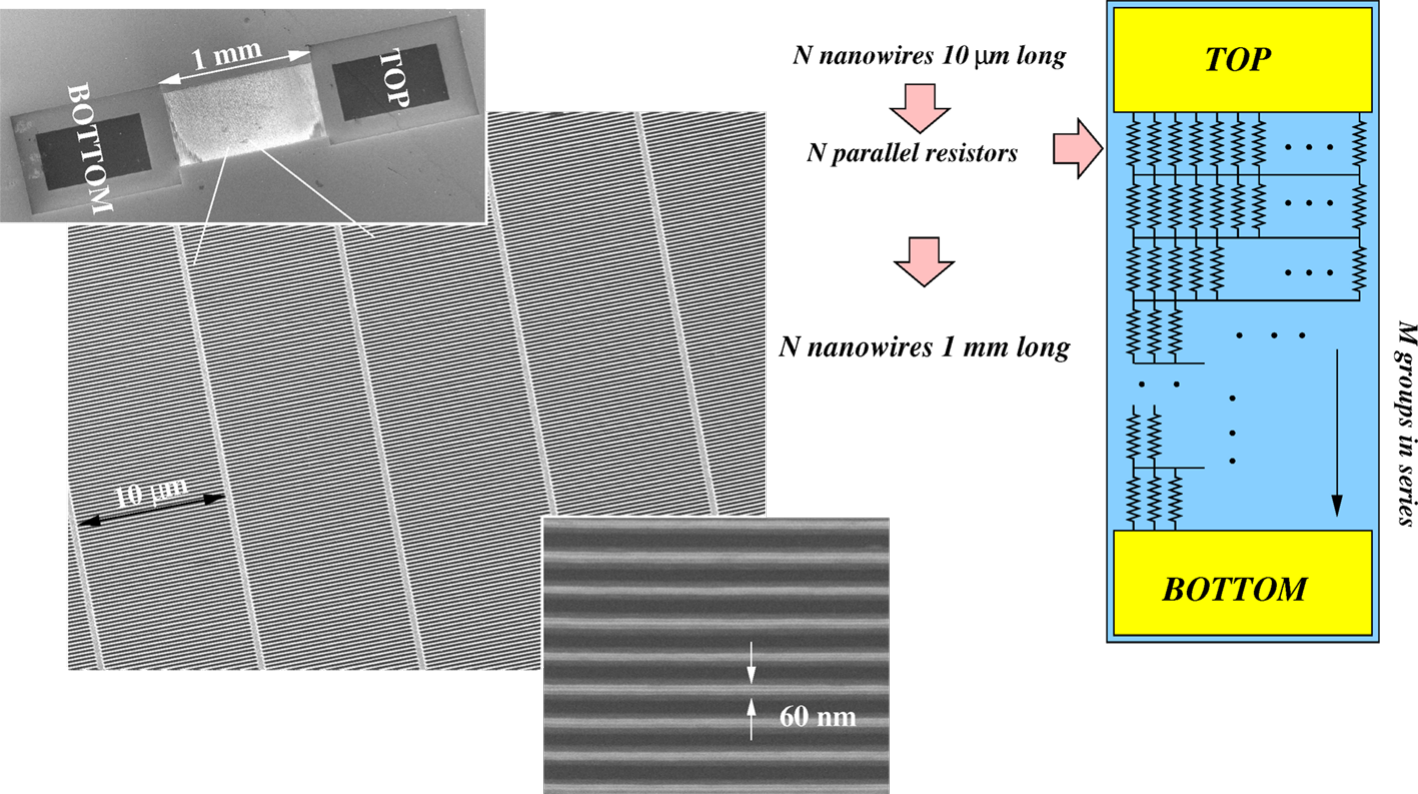
Composition of SEM images of a large area network with a different texture, with respect to Figure 10. An huge number of nanowires, 10 μm long and 60 nm wide, are placed in series and in parallel. The top right sketch shows a resistor network, that represents the silicon nanowire network both from the electrical and from the thermal point of view. Reproduced with permission from ref.[101], Copyright 2013 American Chemical Society.

This top-down technique is very flexible and it can be used for the fabrication of well-organized networks made of a large amount (more than 10^5^ nanowires/mm^2^) of very small nanowires. Figure 9 shows sketches of networks, made of nanowires placed in multiple series and parallels between a top and a bottom contact. Figure 10 and Figure 11 are compositions of SEM images of typical silicon nanowire networks. The nanowires are 3 μm long in Figure 10 and 10 μm long in Figure 11. It is easy to demonstrate [100–101] that these very large area arrays of very narrow, micrometers long, silicon nanowires are equivalent to the parallel arrangement of very narrow SiNWs with a total length of several millimeters (see the top right sketch of Figure 11). These networks can be used as devices for thermoelectric generation because their macroscopic dimensions make it possible to clamp them between a hot and a cold source, as shown in Figure 9. These macroscopic thermoelectric generators are able to exploit the properties shown by materials at the nanoscale, and in particular the reduced thermal conductivity of nanowires. Furthermore, nanowire networks are very reliable with respect to unavoidable failures (breakages) of nanowires, which can happen during the fabrication process or during the operation of the device. This high reliability is due to the high level of interconnections (vertical and horizontal branches) of the net. It has been demonstrated [100] that the total top to bottom electrical resistance of the net increases only negligibly for a nanowire failure percentage up to 40%, that means the failure (breaking) of almost half of the nanowires. The resistance of the net shows also a low sensitivity with respect to the non-uniformity (dispersion) of the nanowire width. The Seebeck coefficient of nanowire networks has been measured [101], and its value resulted in a good agreement with the doping, found by measuring the nanowire electrical conductivity. However, the measured doping concentration was slightly higher than the original doping concentration of the silicon top layer. A reduction of the thermal conductivity in devices based on top-down nanowires has been confirmed by means of a self-heating, self-measuring technique [102]. This technique is based on the principle of 3-ω thermal conductivity measurements [103], already applied to carbon nanotubes [104], and exploits the heat generated in the nanowire by the Joule effect when a current passes through it. The temperature is determined by means of an accurate *I*–*V* measurement, through a suitable model which uses the thermal conductivity as a parameter to be fitted with experimental data.

The main disadvantage of top-down nanowire networks is that they are defined in a very thin layer which must be supported by a substrate. The silicon top layer can be easily detached from the silicon substrate, and positioned on another substrate that must be a good electrical and thermal insulator. Alternatively, with slight modifications to the process, a polysilicon layer deposited on a thermally insulating substrate can be used as starting material. Multiple parallel layers can be positioned and stacked on the same substrate, so that the total current (and electrical power) delivered by the device increases.

**Figure 12:**
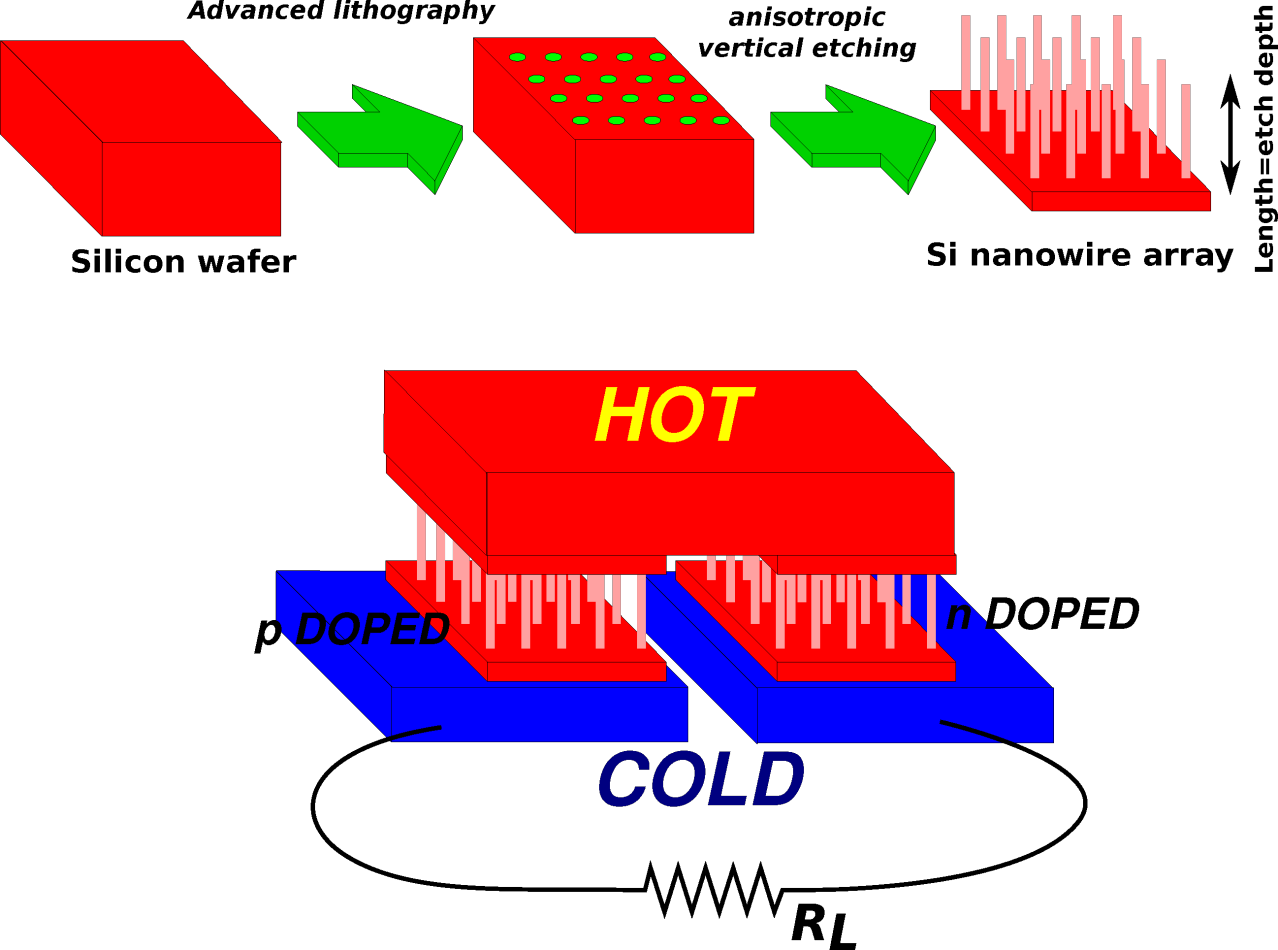
Sketch of the fabrication of vertical nanowire arrays. A sketch of a thermoelectric generator, based on vertical SiNWs, is also shown. Standard silicon doping processes can be used for the fabrication of both n- and p-doped nanowires.

Another top-down technique for the fabrication of silicon nanowires is schematically represented in Figure 12. This technique exploits highly anisotropic silicon etching for the fabrication of dense arrays of nanowires perpendicular to the wafer surface. A suitable mask with nanometric features is patterned on the surface of a standard n- or p-doped crystalline silicon wafer, and then a vertical, highly anisotropic, etching allows for the definition of nanowires. The main advantage of this technique is that a huge number (more than 10^7^ nanowires/mm^2^) of parallel nanowires can be simultaneously fabricated on large surfaces. However, the nanowire length relies on the ability of performing high aspect ratio vertical etches, which is limited not only by the etching selectivity but also by the reduced mechanical stability of very long and narrow nanowires. Deep reactive ion etching (DRIE) is a plasma etching technique that alternates vertical etching steps (for example by SF_6_) and polymerization steps (by CF_4_) [105–108]. Each polymerization step provides a conformal polymer deposition, meanwhile each etching step that follows is anisotropic. Therefore, the etching step removes the polymer on the bottom of the feature while a passivating polymer layer is preserved (or only partially removed) on the sidewalls. In this way, the sidewalls of the features are protected, and the etching proceeds vertically during successive etching and passivation steps. Deep vertical etches can be obtained with a huge number of alternate etching/passivation steps. For its importance and utility also in other fields, such as integrated circuits and the fabrication of micro-electromechanical systems (MEMS), this technique has been improved and developed in the course of the years, with respect to both etching/polimerizating chemical agents and process temperature and step time [106]. Silicon pillars smaller than 100 nm with aspect ratios greater than 50:1 [109] or even 100:1 [110–111] have been fabricated, and thermoelectric power generators based on vertical DRIE fabricated nanowires, few micrometers long, have been tested [112–113].

A very promising technique, which could allow for the fabrication of vertical nanowires with a length of several tens of micrometers, is the silicon metal assisted chemical etching (MaCE) [114–116]. The thermal conductivity of crystalline nanowires, obtained by MaCE, has been measured by Hochbaum and co-workers [9]. In the MaCE technique, a patterned film (or nanoparticles) of noble metals (most commonly gold or silver, but also platinum) on a silicon substrate is used to catalyze the chemical etching of silicon by an aqueous solution of HF and an oxidant as H_2_O_2_. Following the most accepted model, H_2_O_2_ is reduced at the metal through the reaction:

[13]
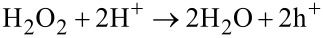


**Figure 13:**
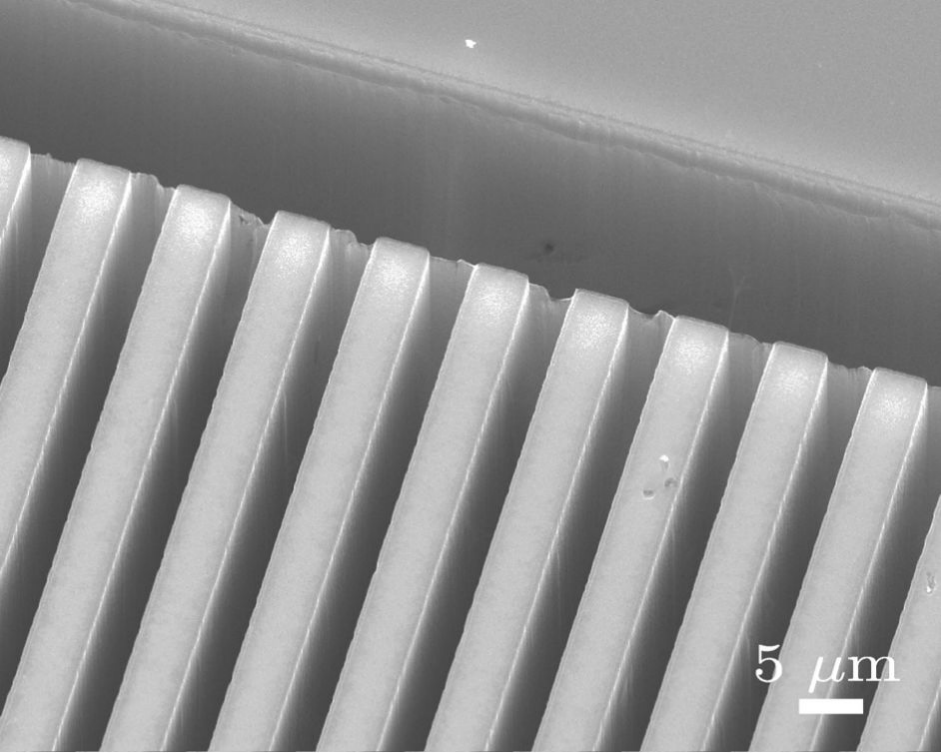
SEM image showing a top view of very deep trenches in silicon, obtained by MaCE. Patterned gold stripes have been used as catalyst.

**Figure 14:**
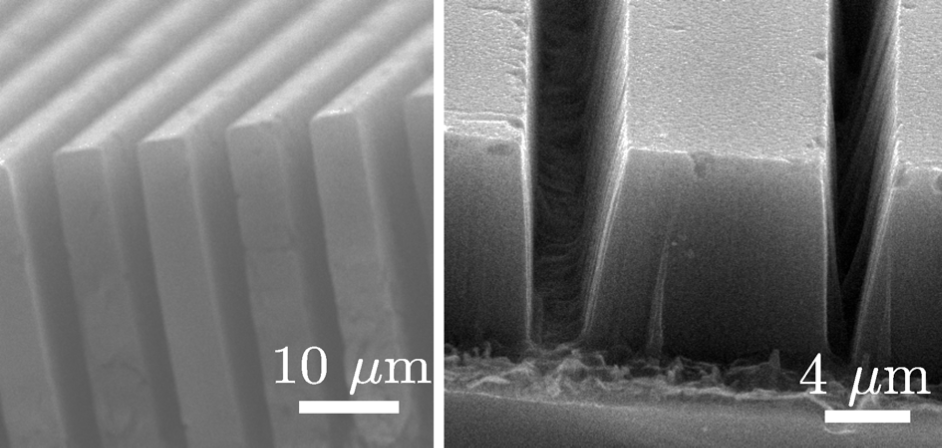
The SEM image on the left shows a cross section of vertical structures, obtained by MaCE etching: Patterned Au structures have been used as a catalyst. The etching solution was HF:H_2_O_2_:H_2_O 3:1:10 in volume. The cross section of the SEM image on the right shows trenches in a silicon substrate obtained by MaCE with an high concentration of hydrogen peroxide (HF:H_2_O_2_:H_2_O 3:3:10). The sidewalls are not vertical, and porous silicon is generated below the metal-free surfaces.

Thus, the metal acts as a microscopic cathode injecting holes in the underlying silicon (i.e., withdrawing electrons from silicon) and the silicon undergoes oxidative dissolution in the presence of HF. Therefore, silicon in direct contact with the metal is dissolved so that the metal sinks into the substrate, and the metal–silicon (Schottky) junction is maintained. In this way, the etching front (metal–silicon interface) moves deeper and deeper into the silicon substrate. Conversely, the silicon etching is very slow in regions without metal coverage, because the electrochemical potential of H_2_O_2_ is much more positive than the silicon valence band energy, and direct injection of holes in silicon is very difficult. Therefore, vertical trenches with high aspect ratio [117–119] (Figure 13) and in particular silicon nanowires narrower than 100 nm and with a length of several micrometers [120–126] can be obtained by using metal masks with suitable patterning. However, if the generation of holes through the reducing reaction (Equation 13) is faster than the silicon oxidation/etching reaction, holes generated at the silicon–metal interface can diffuse toward confining, metal-free, regions [127]. Thus, silicon can become partially etched in neighbouring regions around the metal features [117,124,128] (Figure 14). This limits the minimum distances between metal patterns, as for example the nanowire pitch. Furthermore, hole diffusion in metal-free regions gives porous vertical structures. Porous nanowires must be avoided, because they have a reduced electrical conductivity with respect to bulk silicon, and their *Z* factor is not optimized. Conversely, if the hole generation rate at the metal interface is too low, the etching is not uniform and looses its verticality. In particular, very small metal features as nanoparticles [115,117,119,129] sink into the silicon following random paths, instead of proceeding vertically (perpendicular to the Si surface).

The critical parameter *ε* to be optimized for MaCE is the ratio between the concentrations of HF, which determines the silicon dissolution rate, and H_2_O_2_, which determines the hole generation rate: *ε* = [HF]/[H_2_O_2_] [115] (sometimes the parameter *ρ* = [HF]/([H_2_O_2_] + [HF]) is used instead [119]). The *ε* parameter must be accurately calibrated in order to obtain vertical, and non-porous, structures. If *ε* is too low (high H_2_O_2_ concentration), the etching is more vertical but the silicon surrounding the etched regions becomes porous (porous nanowires). If *ε* is too high, the etching looses its verticality. The correct ε value depends on the crystalline orientation of the silicon wafer and, in particular, on the shape of the metal patterns [115,130]. Values of ε > 3 give random movements of the catalyst particles, while values of ε < 1 give porous structures [124].

Patterns of catalytic metals can be obtained by metal deposition (thermal or e-beam evaporation) and standard lithographic tools. Metal catalyst features for regular silicon nanowire arrays can also be produced by the simpler and cheaper innovative pattering technique nanosphere lithography [120–121]; The evaporation of Ag or Au through porous aluminum oxide membranes[122–124] gives a periodic and almost regular pattern for vertically etched nanowire arrays.

**Figure 15:**
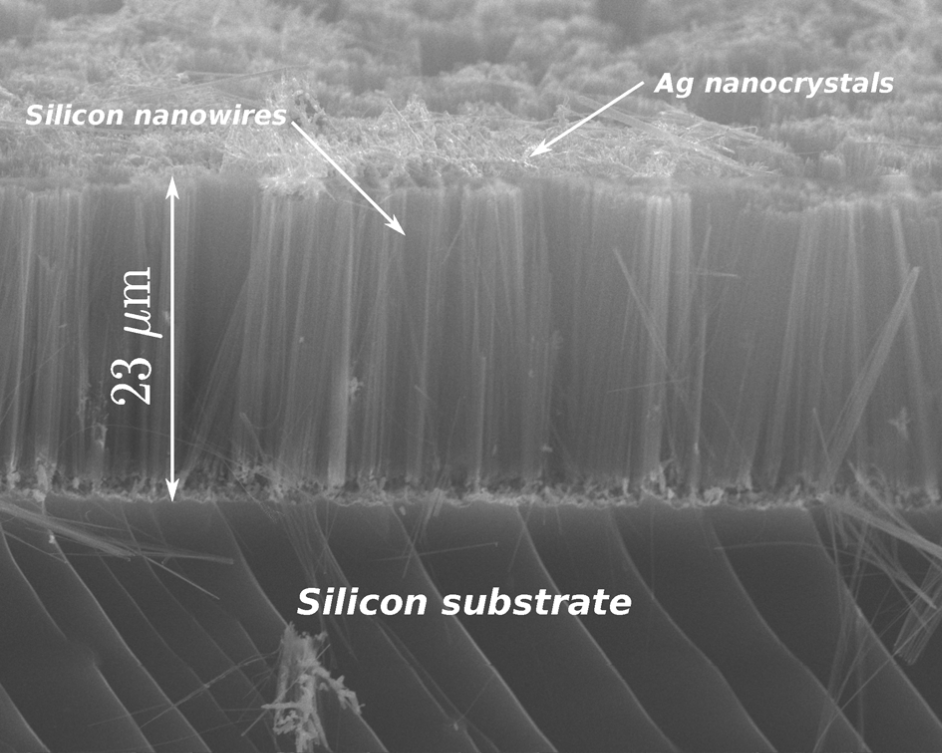
SEM image of a silicon nanowire “forest” (cross-section), fabricated by MaCE of silicon in an HF/AgNO_3_ aqueous solution.

A thin evaporation of a uniform Au film on a silicon surface, with a successive rapid thermal annealing (RTA), can be used for the fabrication of Au nanoparticles on large surfaces. Even if the nanoparticles have a random position and diameter, the average diameter and diameter dispersion can be partially controlled by the initial film thickness and RTA time and temperature [131]. Metal nanoparticles on silicon, suitable for MaCE production of SiNWs, can be also obtained by sinking silicon in an aqueous solution of HF and metal salts, such as AgNO_3_ [132–135], for a very short time (galvanic displacement). Is is to be noted that AgNO_3_ acts as oxidizing agent and it can be used as alternative to H_2_O_2_ for vertical etching. A long etching in a HF/AgNO_3_ solution gives as a result vertical silicon nanowires with random diameter and position. This avoids the need of a preliminary metal pattern deposition and definition. See for example the SEM image of Figure 15, that shows a “forest” of nanowires longer than 20 μm, perpendicular to the Si surface, obtained with a HF/AgNO_3_ based MaCE. The nanowire diameter is in the range of 100 nm. The drawback of this technique is that Ag is continuously deposited during the etching, so that nanoparticles grow and new nanoparticles are continuously deposited on the Si surface. Hence, non-uniform nanowires with a large dispersion in diameter distribution are obtained. However, some results in average nanowire diameter and diameter dispersion control have been obtained [134] by a suitable calibration of the HF/AgNO_3_ concentration ratio and the reaction temperature.

The fundamental point that still needs to be addressed in the development of TEGs based on vertical, large area nanowire arrays is the improvement of techniques for giving them mechanical stability and robustness, and for fabricating a metal structure on the top of the array, to be used as electrical and thermal contact. To these purposes, techniques for embedding vertical silicon nanowire arrays in polymers are under development [136–137]. However, the presence of the polymer limits the maximum working temperature of these TEGs. At the present state-of-the-art, only preliminary, very encouraging, characterizations of vertical silicon nanowire arrays have been performed [136–137] and the Seebeck coefficient has been measured [138].

## Conclusion

In this review paper, after an introduction of the basic principles of thermoelectricity and of the basic parameters for a high TEG conversion efficiency, a quick summary of the most investigated TE materials is given. The aim of research on TE materials is to increase the parameter *Z* = *S*^2^σ/*k*_t_ (or equivalently the figure of merit *ZT*), hence to fabricate TEGs with a high conversion efficiency. In this respect, tellurium compounds seem to be very promising. However, tellurium is very rare on the surface of Earth: It has been estimated that its abundance is only slightly higher than that of platinum. Furthermore, tellurium is poisonous and polluting. Thus, in the case of a large-scale applications of tellurium-based devices their disposal would arise serious environmental problems.

The enhanced phonon surface scattering in rough silicon nanowires can reduce their thermal conductivity by two orders of magnitude (down to few W/mK), with respect to that of bulk silicon (that is 148 W/mK). Conversely, the electrical conductivity of silicon nanowires wider than 20 nm is almost unchanged with respect to bulk electrical conductivity, which is controllable by means of well-known silicon doping processes. Therefore, nanowires offer a very promising way to the use of silicon as TE material, with efficiencies comparable to, and even better than, those of other materials. Silicon is one of the most abundant elements on the surface of Earth, and it is a very sustainable and biocompatible material. Furthermore, silicon-based technologies are largely available and widespread, because their investigation and improvement are stimulated by the continuous growth of the electronic market. For example, the technology of metal contacts on silicon, which are essential for electrical interconnections between the elements of TEG devices, is very well known. Conversely, a full development of solutions and technologies for the fabrication of contacts is requested for many innovative TE materials.

The fabrication of TEGs based on SiNWs must face the challenging fabrication of large scale arrays of SiNW. In this paper, a considerable space has been given to the review of the most innovative technological solutions currently under development for this purpose. Several solutions are very interesting and promising. However, problems of reliability, repeatability on large areas and mechanical stability of very long, and very narrow, silicon nanowires still need to be addressed in future research works.
